# The Analytical Framework of Governance in Health Policies in the Face of Health Emergencies: A Systematic Review

**DOI:** 10.3389/fpubh.2022.628791

**Published:** 2022-06-24

**Authors:** Lina Díaz-Castro, María Guadalupe Ramírez-Rojas, Héctor Cabello-Rangel, Ever Sánchez-Osorio, Mauricio Velázquez-Posada

**Affiliations:** ^1^Direction of Epidemiological and Psychosocial Research, National Institute of Psychiatry Ramon de la Fuente Muñiz (INPRFM), Mexico City, Mexico; ^2^Department of Medical Anthropology, National Council of Science and Technology (CONACYT), Center for Research and Higher Studies in Social Anthropology (CIESAS-Sureste), Chiapas, Mexico; ^3^Research Department, Psychiatric Hospital Fray Bernardino Álvarez, Mexico City, Mexico; ^4^Center and Assistance in Technology and Design of Jalisco State (CIATEJ), Jalisco, Mexico; ^5^Secretary of Health, Mexico City, Mexico

**Keywords:** governance, health policy, pandemic (COVID-19), public policy, government

## Abstract

The Governance Analytical Framework (MAG) defines governance as a social fact, endowed with analyzable and interpretable characteristics, through what it calls observable constitutive elements of governance: the problem, the actors, the social norms, the process of decision-making and scope or nodal points; in the sense that each society develops its modes of governance, its decision-making or conflict resolution systems among its members, its norms, and institutions. In this perspective, the purpose of this article was to carry out a systematic review of the scientific literature to understand the role of governance in health policies in health emergencies, such as that caused by the SARS-CoV-2. The systematic review was designed based on the methodology proposed in the PRISMA (Preferred Reporting Items for Systematic Review and Meta-Analysis) Declaration. The literature search was carried out in six databases: Psychology and Behavioral Sciences, APA-PsycInfo, MEDLINE, eBook Collection (EBSCOhost), PubMED, and MedicLatina, published in the last 5 years. Fifteen articles that met quality and evidence criteria were analyzed. The governance approach alluding to the health emergency problem in health policies was the most addressed by the authors (80%), followed by a description of the actors (40%), the process of decision-making spaces (33%), and ultimately, social norms or rules with 13%. Formulating a coherent set of global health policies within a large-scale global governance framework is mostly absent. Although the countries adopt international approaches, it is a process differentiated by the social, economic, and political contexts between countries, affecting heterogeneous health outcomes over the pandemic.

## Introduction

Health systems worldwide have faced several challenges in meeting one of their primary objectives: service delivery. Regardless of the type of system, structure, organization, and its level of income, one of the shared challenges is related to leadership and governance (1); which refers to the governmental role in public health and its relations with the actors responsible for population health, through the development of strategic policies that respond to the expectations of the environment.

Governance focuses on decision-making and the potential of its actors to subvert national (or international) policy at the local level (2). In this regard, it is essential to understand the process of developing and implementing health policies to address global health emergencies such as the current SARS-CoV-2 pandemic, to generate evidence that serves as the basis for the knowledge of decision-making in the health system's response to face the emergency.

As a generalizable concept, governance refers here to a kind of social facts, formal and informal collective decision-making processes, and the elaboration of social norms concerning public affairs (3). Addressing governance in public health demands to have a delimited, observable, reproducible, and generalizable object. The Governance Analytical Framework (GAF) defines governance as a social fact, endowed with analyzable and interpretable characteristics, through what it calls observable constitutive elements of governance: the problem, the actors, the social norms, the process, and the nodal points (3), in the sense that each society develops its modes of governance, its decision-making or conflict resolution systems among its members, its norms and institutions.

In the present case, to contain the current health emergency, various measures recommended by international organizations have been issued (4), which have adverse effect implications in the different sectors of the population's social and economic development. Besides, governments worldwide have implemented countless health policies in response to the COVID-19 pandemic (5), strategies that require a consensus among decision-makers in health policies. The analysis of the processes of development and implementation of health policies in the face of the current health emergency, from different government levels, will generate substantial evidence in the knowledge of decision-making and how they affect responsibility in health care (6).

However, to date, policymakers have not had access to quality data; it is unknown to what extent implemented policies have mitigated the pandemic and its effects on health outcomes and economic effects (5).

In this perspective, this article's purpose was to conduct a systematic review of the scientific literature to find out what the role of governance has been in health policies in the face of international health emergencies, such as that caused by the SARS-CoV-2 virus.

## Methods

We developed a systematic review and analysis of the international literature published in the last 5 years on the role of governance in health policies addressing health emergencies and specifically in the face of the COVID-19 pandemic.

The literature search period covered from January 1, 2015, to June 30, 2020. The systematic review was designed based on the methodology proposed in the PRISMA Statement (Preferred Reporting Items for Systematic Reviews and Meta-Analyses).

The search of the scientific literature was conducted between April to June 2020 and was carried out in six databases: Psychology and Behavioral Sciences Collection, APA PsycInfo, MEDLINE Complete, eBook Collection (EBSCOhost), PubMED, and MedicLatina. Gray literature was not included.

**Figure 1:**
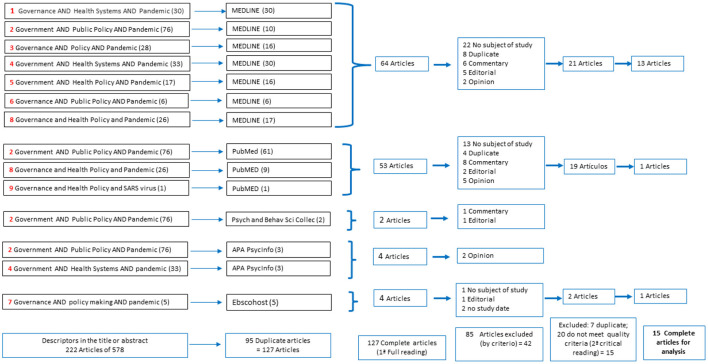
Algorithm for searching the scientific literature of the systematic review.

Following a preliminary review of various terms in the literature and definition of MeSH terms in the databases, the keywords were selected to identify articles relevant to scientists in health policy governance research facing health emergencies and SARS-CoV-2. The search was carried out for 23 combinations of the following descriptors: (1) “governance” or “government”; (2) “health systems” or “organizational policy” or “public policy” or “policy” or “health policy” or “policy-making,” and (3) “SARS virus” or “pandemic.” The descriptor combinations that yielded results are shown in Figure 1.

A total of 578 items were obtained. In the exploration of search terms in the database that include Spanish-language publications, no articles were found.

When narrowing down the search for the terms mentioned in the title and abstract, 222 articles were obtained; in this first filtering, 95 documents were suppressed because duplicates were found.

Titles, summaries, and a full first reading of the 127 articles were examined for content review, under the following inclusion and exclusion criteria:

### Inclusion Criteria

Relevant articles according to our study purpose and level of analysis: (i) approximation to the term of governance in the problem of health policies in the face of health emergencies and SARS-CoV-2; (ii) process and decision-making spaces for health emergencies and SARS-CoV-2; (iii) actors involved in the decision-making; (iv) rules that were adopted for the decision. According to their type, the articles included were research articles, original articles, short research articles, special section, review articles, case studies, author's manuscripts, and journalistic articles.

### Exclusion Criteria

Letters to the editor, news, comments and case report, incomplete articles, and articles that did not include in their approach the study objective of this review were excluded. A total of 42 articles were obtained for an exhaustive, detailed, and critical reading. A checklist was applied according to the Scottish Intercollegiate Guidelines Network (SIGN) adapted form, which assessed the quality and evidence criteria, according to following: (a) sufficient evidence to answer the objective; (b) consistent studies with their conclusions; (c) relevant studies for our objective (similar subject matter); (d) concern about publication bias (origin of studies, groups of researchers, organizations); (e) proposed benefits; (f) feasibility, if the study is applied to the context; (g) recommendations, evidence-based development, and future research. Fifteen articles were excluded for not meeting the proposed quality criteria. Furthermore, seven articles were excluded to be repeated in the PubMed and MEDLINE databases. Finally, 15 articles were included in the systematic analysis (Figure 1).

## Results

Most of the studies reviewed were conducted in the United Kingdom (UK), followed by Asian countries (China 20%, Iran 20%, and Indonesia 7%), from Latin American countries, studies were only reported in Brazil (Table 1). Eighty percentage of the articles were published in the first semester of 2020. Regarding the constituent elements of governance, the governance approach to allude to the problem of health emergency in health policies was the one most addressed by the authors (80%), followed by a description of the participating actors (40%), the process of decision-making and spaces (33%), and ultimately, they addressed social norms or rules of the game with 13% (Table 1).

**Table 1:** Classification of the articles by governance analysis level on health policies in the face of the health emergencies.

	**Chronology of authors**														
**Author/year**	**Connolly (7)**	**Khan et al. (8)**	**Plamondon and Pemberton (9)**	**Aquino et al. (10)**	**Cheng et al. (5)**	**Duan et al. (11)**	**Hsiang et al. (12)**	**Pan et al. (13)**	**Rai et al. (14)**	**Raoofi et al. (4)**	**Requia et al. (15)**	**Shangguan et al. (16)**	**Tabari et al. (17)**	**Taghrir et al. (18)**	**Xu and Yang (19)**
**Source (Journal)**	Disaster	BMC	Health	Cien	Nat	Int J	Nature	Microbes	WHO South	Arch	Sci	Int J	Iran J	Arch	J Epidemiol
	Prev	Public	Res Pol	Saude	Hum	Environ Res		Infect	East Asia J	Iran	Total	Environ Res	Med	Iran	Glob
	Manag	Health	Syst	Colet	Behav	Public Health			Public Health	Med	Environ	Public Health	Sci	Med	Health
**Study Country/Region**	UK	UK	UK	Brazil	UK	Chine	UK	UK	Indonesia	Iran	Brazil	Chine	Iran	Iran	Chine
**Governance analysis level**															
**Country-level coordination, planning, and monitoring**															
Effective communication		X				X	X	X							
Intersectoral participation		X			X	X							X		
Solidarity participation		X			X	X									
Equitable participation		X	X			X									
Responsible governance	X	X		X	X		X			X					
**Risk communication**															
Risk management						X					X	X			X
**Surveillance and rapid-response**															
Epidemiologic surveillance				X	X					X					
**Actors who participate in health policies**															
Multilevel	X	X				X			X						
Multisectoral		X		X					X	X					
Multidisciplinary		X													
**Nodal points**															
Decision making	X	X				X			X					X	
Scope		X							X						
**Rules (or norms in decision making)**															
Formal		X	X												
Informal		X	X												

*The “X” signifies that article contains a category of analysis*.

### Governance Approach in Health Policies in the Face of Health Emergencies

For a better understanding of the study, we divided our analysis of governance in health policies into three critical points identified in the review (Table 1).

#### Country-Level Coordination, Planning, and Monitoring

The studies under analysis demonstrate the national and international scope of the pandemic response (8, 11, 13) and cross-sectoral participation with multi-level representatives with the common goal of generating proactive responses aimed at creating resilient systems (8). The importance of local leadership, ethics, and values of cooperative society (8), incorporating strategies in a coordinated and collaborative manner and integrating equity values (9), reciprocity, protection, self-care, co-responsibility, and solidarity, is underlined (8).

The most widely implemented health policies at the country level are health resources (5) and anti-contagion measures. Policy evaluation studies (12), decision-making process (17), strategic action, or policy design (7) were not identified.

#### Risk Communication and Community Engagement

In the management of the health emergency, first, it is considered (a) disclosure or control of information; (b) hazard and threat assessment; (c) establishment of crisis information communication channels and health education platforms; (d) the development and implementation of strategic response plans, and (e) general mobilization of critical resources (16).

A study evidenced a national public health emergency management system (19) and recommended increasing risk perception in the population, as it is a predictor of public protection measures (11). With this same approach, estimating risks in the design of government intervention policies is an associated strategy (15), pointing out that information control is the basis of health emergency management (16).

#### Surveillance and Rapid-Response Teams, and Case Investigation

Conceived as one of the essential functions of public health, the epidemiological surveillance policies (20) adopted by the member countries, follow the context, the preparation of health facilities, financing, health personnel, information and research, and medical products and technologies (4). Most governments have implemented policies in response to COVID-19 that are restrictions on external (border closure) and internal (school closure) mobility. The response involves various political and technical decisions; a study reported how limiting the response was based only on health services' capacity and not on a consensus to follow international recommendations (10).

The next element of governance under study refers to actors, involving two hierarchically and relationally in power dynamics (21).

### Actors Involved in Health Policies in the Face of Health Emergencies

In the design and management of public health policies, a multi-level perspective is incorporated, this implies the participation of multiple actors (7) at the international level, such as the WHO and the Global Health Security Agenda (GHSA), actors in the government system, from the Federal Government, Municipal Governments, the Judiciary, the Legislative Branch, and the Ministry of Health; and Community actors (11, 14). Various interests of actors or groups seeking solutions converge in decision-making, even from their belief system (4). A study identified that those responsible for implementing, monitoring, and evaluating the response to the emergency (14) incorporated multisectoral coordination mechanisms, active participation of all stakeholders, and presidential support. Another successful study incorporated high-level decision-making, experts in preparing health emergencies (8). It was also documented that the lack of consensus among the different actors limits the effectiveness of the response (10).

### Process of Decision-Making and Scope in Decision (Nodal Points)

Addressing the health emergency problem scenarios is complicated due to diverse interests in decision-making (7). Few studies document how the different actors participate, interrelated strategic levels of action in epidemic management and policy design (14). However, they demonstrate the international scope of responding to pandemics (8, 11, 22) at a high-level decision-makers and the need to assess all political decisions' success and failure to find the appropriate course of action in the high-level response (18).

### The Rules of the Game (Normative, Formal, and Informal)

Finally, the WHO regulatory framework guides strategies; however, effective responses have documented the importance of local leadership, ethics and values, implying a set of formal and informal rules (8) in a coordinated, collaborative way and incorporating equity values (9). In other words, to make ethical decisions, it is necessary to include processes of inclusion, accountability, transparency, and responsiveness (8).

## Discussion

On January 30, 2020, the WHO Director-General declared the outbreak of the coronavirus disease 2019 (COVID-19) as a public health emergency of international importance under the International Health Regulations (23). On February 4, 2020, the WHO requested the United Nations crisis management policy's activation to establish a Crisis Management Team to help member countries to prepare for and respond to the emergency (23).

### The Problem From Governance

The globally rapid spread of COVID-19 has created and exhibits a wide range of nuances and heterogeneity of health policies implemented by governments (24), making it difficult to assess them (25) to adopt it and hinders its recommendation, which shows the absence of a global governance framework (26). Despite this, most countries' governance approach follows the policies or measures suggested by the WHO (4) and the United Nations' strategic response and preparedness plan for COVID-19 (27). Countries like China and Canada have reported success in controlling the pandemic; however, in less developed nations, the persistence of health inequities has been a problem formed by the power systems themselves, in which competing social interests and values further increase these inequities (9).

The policies put into practice must be evaluated to address the response and solutions adopted to the pandemic. Nevertheless, in a study it is describe that the (international) response has been effective in containing the pandemic, it does not detail the decision-making process (17), nor monitoring activities (or indicators) for overall policy evaluation. Therefore, it is not possible to establish the extent to which the policy is effective or the scope achieved, or what information is required to measure that policy (28), and in any case, redesign it.

### The Actors

Decisions in health policies in the face of health emergencies involve various actors, from the international scope, governmental at all levels, and community actors (11, 14). The particular interests of these actors converge on the political decision-making process. In fact, they can seek solutions based on their belief system; this phenomenon cannot be set aside in decisions, but an objective process must be included into the decision, for example, incorporating a coalition political system to achieve agreements between the participating actors and an evaluation on the implementation performance of the resulting policies (4). Therefore, to ensure that the policy's implementation is effective, it is essential to document the monitoring and evaluation of the response to the COVID-19 emergency (14), including all the multi-sector coordination mechanisms achieved among actors, as well as the active participation of all stakeholders (8). As documented, when agreements are partial or unilateral, the response's effectiveness is limited; in fact, the lack of consensus between the different actors leads to adverse health outcomes (10).

### The Process of Decision-Making and Scope in Decision

The mechanisms and scopes of participation in the different strategic action levels related to epidemic management and policy design need to be made visible and documented (7), to adopt significant pandemic control recommendations. Beyond the global stage, policy success lies in local capacity to subvert them. In this sense, there are differential effects between categories of government intervention and public adoption of measures in communities. In this scenario, it is recommended to increase the risk perception in the population, as government actions are related and predict public protection measures (11). An example of an associated government strategy is risk estimation in designing intervention policies (15).

On the other hand, dissemination and control of information in the health crisis are the basis in the design of management policies to face the emergency. Similarly, the scientific assessment of the emergency is necessary for the subsequent formulation of intervention policies; it must be based on accurate information; otherwise, the crisis can expand negatively (16). In this sense, the Chinese government published a success report, which has already established a national public health emergency management system (19).

Regarding the rules of the game (social rules), although international standards guide decisions in response to pandemics anywhere, to generate effective responses, local leadership, ethics, and social values are paramount, implying a game of formal and informal rules, including all society sectors (8) in a coordinated, collaborative way; it must also incorporate values like equity, reciprocity, trust, public protection, self-care, co-responsibility, and solidarity.

Therefore, the establishment of a global health governance framework that ensures equitable access for all to adequate health care in health emergencies should be in a prominent place on the global policy and legislative agenda. Though, the formulation of a coherent set of global health policies on a large scale is largely absent.

The literature discussed here was made in socially and economically developed nations, which have actors with some decision-making power (29) in international policies, as well as in the design of their indicators in the health system (30); but in those economically disadvantaged countries, with great social inequality, with a lower budget and health spending, with a weak structure of health systems (31), there are more significant disadvantages to adopting international recommendations to address pandemics. Another challenge for governance in these countries' health policies is that governments should consider local peculiarities, viability, sustainability, and potential risks and benefits before and after of public health policies implementation (32).

In this differentiated context of policies for protection and response to threats and vulnerabilities, from national and international guidelines, the necessity for countries to incorporate academics and civil society leaders at the local level is seen to integrate their perspective into the response to the health needs of the population (33). It is crucial to implement and document risk management policies, which implies the acquisition of an empirical response to an accelerated and rapidly changing dynamics of the COVID-19 pandemic.

A potential limitation to our analysis is the lack or scarcity of research on this topic, especially in less developed countries, thus, our findings may not include considerations from other countries not represented in the literature reviewed. On the other hand, we do not use gray literature, therefore, it is possible that we may have missed relevant information about the practice on governance in the health systems reported in this type of literature. Despite these limitations, we think that this study serves to demonstrate the need to increase the evidence on governance in health systems to face health emergencies.

## Conclusions

This systematic review from a GAF approach allowed us to analyze governance challenges and its current state to subvert them from the international level to local scenarios in order to implement risk management policies. In future research, the GAF could be applied to identify and incorporate the analysis of other social actors with different levels of decision-making to respond to health emergencies. This could be documented to adapt them in different contexts.

## Author Contributions

LD-C and GR-R contributed the design, data analysis, interpretation, and writing of first and subsequent drafts of the paper. HC-R, ES-O, and MV-P contributed data analysis, interpretation and writing of first drafts of the paper. All authors contributed to the article and approved the submitted version.

## Funding

Our research was funded by the National Council of Science and Technology (CONACYT, México), Project # 313274.

## Conflict of Interest

The authors declare that the research was conducted in the absence of any commercial or financial relationships that could be construed as a potential conflict of interest.

## Publisher's Note

All claims expressed in this article are solely those of the authors and do not necessarily represent those of their affiliated organizations, or those of the publisher, the editors and the reviewers. Any product that may be evaluated in this article, or claim that may be made by its manufacturer, is not guaranteed or endorsed by the publisher.
